# Candidate gene study of *HOXB1 *in autism spectrum disorder

**DOI:** 10.1186/2040-2392-1-9

**Published:** 2010-05-25

**Authors:** Lucia A Muscarella, Vito Guarnieri, Roberto Sacco, Paolo Curatolo, Barbara Manzi, Riccardo Alessandrelli, Grazia Giana, Roberto Militerni, Carmela Bravaccio, Carlo Lenti, Monica Saccani, Cindy Schneider, Raun Melmed, Leonardo D'Agruma, Antonio M Persico

**Affiliations:** 1 Medical Genetics Service, IRCCS 'Casa Sollievo dalla Sofferenza', San Giovanni Rotondo, FG, Italy; 2 Laboratory of Molecular Psychiatry and Neurogenetics, University 'Campus Bio-Medico', Via Alvaro del Portillo 21, I-00128 Rome, Italy; 3 Laboratory of Molecular Psychiatry and Psychiatric Genetics, Department of Experimental Neurosciences, IRCCS 'Fondazione Santa Lucia', Rome, Italy; 4 Department of Child Neuropsychiatry, University of Rome 'Tor Vergata', Rome, Italy; 5 Department of Child Neuropsychiatry, II University of Naples, Italy; 6 Department of Pediatrics, University 'Federico II', Naples, Italy; 7 Department of Child Neuropsychiatry, University of Milan, Milan, Italy; 8 Center for Autism Research and Education, Phoenix, AZ, USA; 9 Southwest Autism Research and Resource Center, Phoenix, AZ, USA; 10 Current Address: Laboratory of Oncology, IRCCS 'Casa Sollievo della Sofferenza', San Giovanni Rotondo (FG), Italy

## Abstract

**Background:**

*HOXB1 *plays a major role in brainstem morphogenesis and could partly determine the cranial circumference in conjunction with *HOXA1*. In our sample, *HOXA1 *alleles significantly influence head growth rates both in autistic patients and in population controls. An initial report, suggesting that *HOXB1 *could confer autism vulnerability in interaction with *HOXA1*, was not confirmed by five small association studies.

**Methods:**

Our sample includes 269 autistic individuals, belonging to 219 simplex and 28 multiplex families. A mutational analysis of the two exons and flanking intronic sequences of the *HOXB1 *gene was carried out in 84 autistic patients by denaturing high performance liquid chromatography, followed by DNA sequencing. Identified rare variants were then searched by a restriction analysis in 236 autistic patients and 325-345 controls. Case-control and family-based association studies were performed on two common variants in 169 Italian patients versus 184 Italian controls and in 247 trios.

**Results:**

We identified three common polymorphisms, rs72338773 [c.82insACAGCGCCC (INS/nINS)], rs12939811 [c.309A>T (Q103H)], and rs7207109 [c.450G>A (A150A)] and three rare variants, namely IVS1+63G>A, rs35115415 [c.702G>A (V234V)] and c.872_873delinsAA (S291N). SNPs rs72338773 and rs12939811 were not associated with autism, using either a case-control (alleles, exact *P *= 0.13) or a family-based design [transmission/disequilibrium test (TDT)χ^2 ^= 1.774, *P *= 0.183]. The rare variants, all inherited from one of the parents, were present in two Italian and in two Caucasian-American families. Autistic probands in two families surprisingly inherited a distinct rare variant from each parent. The IVS1+63A allele was present in 3/690 control chromosomes, whereas rare alleles at rs35115415 and c.872_873delinsAA (S291N) were not found in 662 and 650 control chromosomes, respectively. The INS-T309 allele influenced head size, but its effect appears more modest and shows no interaction with *HOXA1 *alleles. The INS-T309 allele is also associated with more severe stereotypic behaviours, according to ADI-R scores (*N *= 60 patients, *P *< 0.01).

**Conclusions:**

*HOXB1 *mutations do not represent a common cause of autism, nor do *HOXB1 *common variants play important roles in autism vulnerability. *HOXB1 *provides minor, albeit detectable contributions to head circumference in autistic patients, with *HOXA1 *displaying more prominent effects. *HOXB1 *variants may modulate the clinical phenotype, especially in the area of stereotypic behaviours.

## Background

Genetic contributions to autism have received strong support from family and twin studies [1,2]. A relatively small number of major loci was initially predicted to explain the disease in the majority of affected individuals [2]. However, the number of common genetic variants conferring vulnerability to autism has grown well beyond the initial expectations, in addition to several rare variants identified to this date, each explaining the disease in a very small number of patients [[3-5]. The genetic underpinnings of autism spectrum disorders have thus proven to be far more complex than expected, likely due to genetic heterogeneity, epistasis and gene-environment interactions [3-5].

Several lines of evidence have demonstrated altered prenatal neurodevelopment as central to autism pathogenesis. Abnormal neurodevelopment possibly occurring in the first trimester of pregnancy best accounts for the microscopic alterations shown by *post-mortem *neuroanatomical studies of the brains of autistic patients [6,7]. Phenotypic evidence also supports a prenatal aetiology, as fine motor abnormalities are detectable at 4-6 months of age or even at birth in children later developing autism [8]. Finally, a prenatal time window as early as days 20-25 post-fertilization appears crucial to autism's aetiology, since exposure to thalidomide during pregnancy leads to autism only if occurring within this limited developmental interval [9]. Genes encoding proteins involved in early neural development could thus encompass polymorphisms or mutations contributing to the disease process.

The *HOXB1 *gene, located on human chr 17q21.32, is a member of the HOX gene family of homeobox transcription factors and critically involved in the development of the brain stem. *HOXB1 *gene expression is limited to rhombomere 4 and to neural crest cells migrating out of this region, resulting in a gross reduction or complete loss of the facial motor nucleus in *HOXB1 *mutant mice [10,11]. A similar loss of facial motor neurons has been described in one autistic brain [12]. *HOXB1 *gene expression occurs very early in mouse development (E8.5-E9.5) [10,11], at a time, interestingly, overlapping with the window of maximal prenatal sensitivity in rodent models of autism [13]. *HOXB1 *gene expression is also strongly up-regulated by *HOXA1 *[14] and the two genes indeed synergize in patterning hindbrain structures, cranial nerves and pharyngeal arches, so that double-mutant mice display prominent malformations while single mutants suffer much milder abnormalities [14-16]. A gene × gene interaction between *HOXB1 *and *HOXA1 *gene variants was initially proposed to contribute to autism [17]. In our sample, the *HOXA1 *c.218A>G [His73Arg] polymorphism was significantly associated with autism, although we found an association with the A218 and not the G218 allele [18], in contrast to the original study [17]. Moreover, *HOXA1 *c.218A>G alleles were found to significantly influence head growth rates, and not final head size, both in autistic patients and in typically developing children [18,19]. The latter result is especially interesting, considering that approximately 20% of autistic patients consistently show macrocephaly and may represent an endophenotypic subgroup possibly sharing common pathophysiological underpinnings [20,21]. Following the initial positive study [17], five studies subsequently reported negative association findings using *HOXB1 *gene markers [22-26]. However, the sample sizes assessed in all of these studies were too small to detect moderate-size effects and rare variants of potential clinical relevance (see Discussion). Furthermore, only two of these studies apparently performed mutational searches by DNA sequencing [22,23]. The present study was thus undertaken to screen a larger sample of autistic patients, unaffected controls and nuclear families for *HOXB1 *gene mutations, rare variants and common polymorphisms, either causing the disorder, conferring autism vulnerability, influencing the clinical expression of the disease, or modulating cranial growth rates by themselves or in interaction with *HOXA1 *gene variants.

## Methods

### Subjects

Families were recruited for this study based on the presence of a proband diagnosed with primary autism spectrum disorder (idiopathic, non-syndromic ASD). The clinical sample includes 269 autistic individuals and 593 first-degree relatives, belonging to 219 simplex and 28 multiplex families. In reference to ethnicity, these families encompass 171 simplex and one multiplex Italian families, as well as 48 simplex and 27 multiplex Caucasian-American families, the latter including 15 simplex and 23 multiplex families obtained from the Autism Genetic Resource Exchange (AGRE) repository. Demographic and clinical characteristics of the autistic patients are summarized in Table 1. In addition, we also recruited 345 normal adult controls (M/F ratio = 2.43; mean age ± standard deviation = 33.1 ± 9.2 years, range 18-53), among blood donors at the Haematology Unit of 'Casa Sollievo della Sofferenza' hospital (S Giovanni Rotondo, FG, Italy) and self-reporting no history of major psychiatric disorders or psychotropic drug use for at least 6 months prior to blood donation. This clinical sample largely overlaps with the sample assessed in our previous works on the *HOXA1 *gene [18,19]. The size of subgroups of patients and families involved in the different studies described in the present report are summarized in Additional File 1: Table S1.

**Table 1:** Demographic and clinical characteristics of the autistic patients (*N *= 269, unless otherwise specified).

		Mean/median	Standard deviation	Range
*Mean Age (year)*		**9.3**	**5.6**	**3-33**

*Median VABS scores *(N = *149*)				

	*Communication*	**71**		**19-112**

	*Daily living skills*	**65**		**19-107**

	*Socialization*	**66**		**20-103**

	*Motor skills*	**80**		**37-114**

	*Composite*	**60**		**19-103**

		**N**	**(%)**	

*Sex*				

	*Male*	**242**	**(90.0)**	

	*Female*	**27**	**(10.0)**	

	*M/F ratio*	**9.0:1**		

*Family type*				

	*Simplex*	**219**	**(88.7)**	

	*Multiplex*	**28**	**(11.3)**	

*DSM-IV diagnosis*				

	*Autistic disorder*	**208**	**(77.3)**	

	*Asperger syndrome*	**22**	**(8.2)**	

	*PDD-NOS*	**39**	**(14.5)**	

*Intellectual quotient *(N = *99*)				

	*>70*	**38**	**(38.4)**	

	*≤ 70*	**61**	**(61.6)**	

VABS, Vineland Adaptive Behavior Scales; DSM, Diagnostic and Statistical Manual of Mental Disorders; PDD-NOS, pervasive development disorder not otherwise specified.

Inclusion criteria and diagnostic screening methods have been previously reported [18,19,21] . Briefly, patients fulfilling the Diagnostic and Statistical Manual of Mental Disorders (DSM)-IV diagnostic criteria for autistic disorder, Asperger's disorder or pervasive developmental disorder not otherwise specified (PDD-NOS) [27] were screened for non-syndromic autism using magnetic resonance imaging, electroencephalogram, audiometry, urinary aminoacid and organic acid measurements, cytogenetic and fragile-X testing. Patients with dysmorphic features were excluded, even in the absence of detectable cytogenetic alterations. Patients with sporadic seizures (< 1 every 6 months) were included; patients with frequent seizures or focal neurological deficits were excluded. Autistic behaviours were assessed using the official Italian version of the Autism Diagnostic Observation Schedule (ADOS) and the Autism Diagnostic Interview-Revised (ADI-R) [28,29], available since 2005; adaptive functioning was assessed using the Vineland Adaptive Behavior Scales; intellectual quotient was determined using either the Griffith Mental Developmental Scales, the Coloured Raven Matrices, the Bayley Developmental Scales or the Leiter International Performance Scale. Head circumference was measured in all ASD patients aged <16 years old and transformed into percentiles using the sex- and age-specific standard tables currently adopted in the vast majority of European countries and by the Italian Pediatric Association, as described [21]. This study, as well as the consent form signed by all parents for themselves and their children, was approved by the Institutional Review Board (IRB) of the University Campus Bio-Medico (Rome, Italy). Controls provided written informed consent to the use of their biomaterials for scientific research, using the consent form approved by the IRB of 'Casa Sollievo dalla Sofferenza' hospital (S Giovanni Rotondo, FG, Italy).

### Primer design and polymerase chain reaction (PCR) amplification conditions

PCR primers were designed to amplify the two exons of the *HOXB1 *gene (Ensembl: ENST00000239174) and exon-intron boundaries, extending 30-100 bases into intronic sequences both 5' and 3' of each exon. Primers are described in Table 2. PCR amplifications were performed using a Gene Amp PCR System 9700 (Perkin Elmer, CA, USA) in a final reaction volume of 25 μL containing 100 ng of genomic DNA template, 250 μM dNTPs, 0.5 μM of each primers, 1.25 U Ampli taq GoldTM DNA polymerase (PE Applied Biosystems, CA, USA), in 1X reaction Buffer (10 mM Tris HCl pH 8.3, 50 mM KCl, 2.5 mM MgCl2). PCR cycling conditions consisted of an initial denaturation step at 95°C for 12 min, followed by 35 cycles of 95°C for 30 s, annealing at temperatures reported in Table 2 for 30 s and elongation at 72°C for 10 min.

**Table 2:** Primer sequences for *HOXB1 *gene PCR amplification and DHPLC analysis.

Exon	Primer name	Primer sequences (5'-3')	Amplicon size (bp)	PCR annealing temp (°C)	DHPLC oven temp (°C)
1	HOXB1-F1 for	CATACTGCCGAAAGGTTGTAG	365	60	61.5, 65.6, 66.2

	HOXB1-F1 rev	TAGTACTGAGAAGGCCCGTA			

					

1	HOXB1-F2 for	GGTATGCTCCTGCCGCCTGCA	227	58	63.3, 65.5

	HOXB1-F2 rev	ATCAGCATAGGCCGGTGCAA			

					

1	HOXB1-F3 for	AGCATCCCCCTTATGGGAA	282	58	62.8

	HOXB1-F3 rev	CTTACCTGTGTCTACCAGAG			

					

2	HOXB1-F4 for	GAGAATTGACCTGGCCTTTC	359	60	62.9, 63.4

	HOXB1-F4 rev	TGACAGAGCTGGGTGAGGCTT			

					

2	HOXB1-F5 for	TTTGGTTCCAGAACCGACGA	300	60	64.0, 65.5

	HOXB1-F5 rev	GGCAGCTCTAAACTGGACTT			

PCR, polymerase chain reaction; DHPLC, denaturing high performance liquid chromatography; temp, temperature.

### Wave^® ^System denaturing high performance liquid chromatography (DHPLC) analysis

PCR products from each patient and one normal control were mixed, denaturated for 5 min at 95°C and cooled slowly for 30 min down to 40°C in a thermal cycler, to enable the formation of heteroduplexes. Analytical conditions for each fragment were determined using the Transgenomic WaveMaker™ Software v.4.1.44 (Transgenomic Inc, NE, USA). Samples were run on the 3500HT Wave™ DNA Analysis System (Transgenomic). PCR amplicons were loaded (5 μL) on a C18 reverse-phase column based on nonporous poly (styrene/divinil-benzene) particles (DnaSep™ column; Transgenomic). Hetero and homoduplex analysis was carried out with an acetonitrile gradient formed by mixing buffer A (0.1 M TEAA) and buffer B (0.1 M TEAA, 25% acetonitrile). Flow-rate was 0.7 mL/min with an increase of buffer B of 2% per min for 4.5 min and DNA was detected at 260 nm. All samples showing an abnormal elution profile were sequenced.

### Sequence analysis

PCR products of patients yielding abnormal chromatograms were purified using GFXTM PCR DNA and Gel Band Purification Kit (Amersham Biosciences, CA, USA). DNA sequencing was performed in a 10 μL final volume, with 3 pmol of primer, 4-6 ng of DNA template and 2 μL of Big Dye Terminator Ready Reaction mix v. 2.1 (PE-ABI, CA, USA), using an ABI PRISM 3100 Genetic Analyser, v.3.7 (PE Applied Biosystems, Foster City, CA). DNA sequence analysis was performed using Sequencing Analysis program v. 3.7 (PE Applied Biosystems).

### Genotyping

SNP rs12939811 (Q103H) was genotyped by PCR amplification and DHPLC analysis (temperature melting 63.3°C). In order to discriminate homozygosity for the common allele (AA) from homozygosity for the rare allele (TT), the samples resulting in a single elution profile were mixed with a control sample carrying the AA genotype and were analysed again by DHPLC under at the same temperature conditions. All samples compatible with a TT genotype were then sequenced for confirmation. SNPs rs72338773 (c.82insACAGCGCCC), IVS1+63G>A, S291N and rs35115415 (V234V) were each genotyped by PCR amplification and allele-specific restriction analysis. Primer sequences for *HOXB1 *genotyping are listed in Table 3. Briefly, restriction analyses were performed, as follows: (a) rs72338773 (c.82insACAGCGCCC) was amplified using primers HOXB1-F1_for/HOXB1-F1_rev; the 365 bp fragment was digested with *Msp*I, yielding 140, 121 and 113 bp fragments in the presence of the insertion; (b) the IVS1+63G>A SNP was amplified with primers IVS1+63G>A-mut_for/IVS1+63G>A_rev and the 118 bp amplicon was digested using *Hph*I, producing two 83 and 35 bp fragments in the presence of the A allele; (c) the c.872_873delinsAA (S291N) SNP was amplified using primers S291N-mut_for/HOXB1-F5_rev and the 366 bp fragment was digested with *Mnl*I, yielding 186, 112bp, 50bp and 18bp fragments with the N allele; and (d) rs35115415 (V234V) was amplified using primers S291N-mut_for/HOXB1-F5 rev and the 366 bp fragment was digested with *Fok*I, yielding 335 and 31 bp in the presence of the A allele.

**Table 3:** Primer sequence for *HOXB1 *SNP genotyping.

Exon	Primer name	Primer sequences (5'-3')	Amplicon size (bp)	PCR annealing temp (°C)
1	HOXB1-F1 for	CATACTGCCGAAAGGTTGTAG	365	60

	HOXB1-F1 rev	TAGTACTGAGAAGGCCCGTA		

				

1	HOXB1-F2 for	GGTATGCTCCTGCCGCCTGCA	227	58

	HOXB1-F2 rev	ATCAGCATAGGCCGGTGCAA		

				

1	IVS1+63 G/A-mut_for	AAAGCATCTCTGCTTCCCCTGCGG	118	58

	IVS1+63G/A_rev	GACCTCACCTGACCTGAGAC		

				

2	S291N-mut_for	ACCTGAGCCGGGCCCGGAGGATG	366	58

	HOXB1-F5 rev	GGCAGCTCTAAACTGGACTT		

PCR, polymerase chain reaction; temp, temperature.

### Data analysis

Hardy-Weinberg analyses were performed using the Hardy-Weinberg equilibrium (HWE) and HWE2 programs [30]. Case-control allelic and genotypic distributions were contrasted applying the χ^2 ^statistics after randomly selecting one patient per multiplex family. Single-marker and family-based association analyses were performed applying the transmission/disequilibrium test (TDT) [31] using the TDTPHASE software of the UNPHASED package [32]; only complete trios and one trio per multiplex family were included in these analyses. All family-based association analyses were carried out on Italian and Caucasian-American families merged together after population structure analyses provided no evidence of genetic dyshomogeneity in a subgroup of 179 autistic patients including 155 Italians and 24 Caucasian-Americans randomly chosen one per family, genotyped at 90 unlinked SNPs distributed genome-wide and analysed using the STRUCTURE program [33]. Since this stratification analysis did not include unaffected controls, case-control contrasts employed only individuals of Italian ancestry. Power analyses were performed using P^2^BAT [34]. The phylogenetic conservation of sequences encompassing *HOXB1 *polymorphisms was assessed by orthologue sequence alignments performed using the ClustalW2 [35] and VISTA [36] softwares. The distributions of cranial circumference percentiles in cases and controls were contrasted using the Mann-Whitney U test and the Moses test of extreme reactions, to analyse differences in central tendency and dispersion, respectively [37]. Data are expressed as mean ± standard error of mean, except for the head circumference which is expressed as median ± interquartilic range. Two-tail *P *values are reported throughout the manuscript. The outcome of analyses with phenotypic variables (items of behavioural scales, patient and family history variables and head circumference) underwent three levels of Bonferroni correction for multiple testing: (a) 'stringent', assuming each phenotypic variable as independent: *P *= 0.05/41 = 0.0012; (b) 'intermediate', counting as a single entity only those items which record clearly overlapping phenomena (for example, 'presence of motor stereotypes' in patient history and ADI-R item C): *P *= 0.05/28 = 0.0018; and (c) 'relaxed', considering all variables, except for the head circumference, as non-independent entities clustered into four principal components, in accordance with our recent work [38]: *P *= 0.05/5 = 0.01.

## Results

### HOXB1 gene variants

The mutational analysis performed by DHPLC and DNA sequencing in 84 autistic patients unveiled three common polymorphisms and three rare variants. The common polymorphisms, which were all previously described [17,22,39], are: (a) rs72338773 [c.82insACAGCGCCC (INS/nINS)], here also named c.82ins9; (b) rs12939811 [c.309A>T (Q103H)]; and (c) rs7207109 [c.450G>A (A150A)]. The 9-bp insertion c.82insACAGCGCCC was initially reported by Faiella *et al. *[39] and introduces into the amino acid sequence the tripeptide H-S-A. Two of the three common variants, namely rs72338773 and rs12939811, were genotyped in 169 ASD patients and 184 controls, all of Italian ethnicity. Genotypic and allelic distributions are presented in Table 4.

**Table 4:** Case-control study for the *HOXB1 *polymorphisms rs72338773 [c.82insACAGCGCCC (INS/nINS)] and rs12939811 [c.309A>T (Q103H)].

Genotypes	Italian patients (*N *= 169)	Italian controls (*N *= 184)	Alleles	Italian patients (*N *= 338)	Italian controls (*N *= 368)
**nINS/nINS**	100 (59.2%)	125 (67.9%)	**nINS**	262 (0.7751)	303 (0.8234)

**nINS/INS**	62 (36.7%)	53 (28.8%)	**INS**	76 (0.2249)	65 (0.1766)

**INS/INS**	7 (4.1%)	6 (3.3%)			

	χ^2 ^= 2.93, 2 df, *P *= 0.23, ns			Exact 2-tail *P *= 0.13, ns	

**A/A**	102 (60.4%)	125 (67.9%)	**A**	263 (0.7781)	304 (0.8261)

**A/T**	59 (34.9%)	54 (29.3%)	**T**	75 (0.2219)	64 (0.1739)

**T/T**	8 (4.7%)	5 (2.8%)			

	χ^2 ^= 2.61, 2 df, *P *= 0.27, ns			Exact two-tail *P *= 0.13, ns	

INS, insertion; ns, not significant.

**Figure 1:**
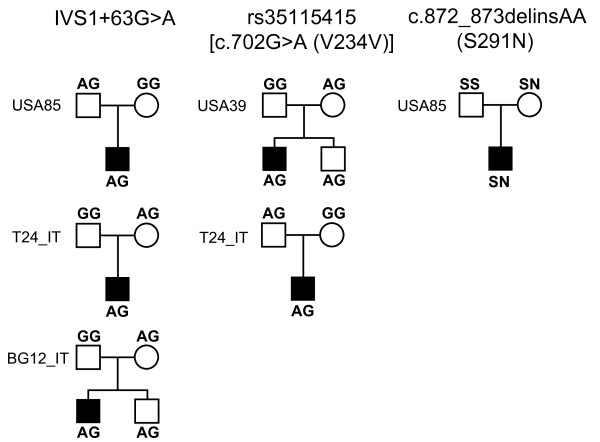
**Pedigrees of families carrying rare variants in the *HOXB1*gene**.

The three rare variants - including (a) IVS1+63G>A, (b) rs35115415 [c.702G>A (V234V)] and (c) c.872_873delinsAA (S291N) - were searched in a total of 236 autistic patients. In all cases, rare variants were inherited by the proband from one of the parents, as depicted in Figure 1. Allelic frequencies are as follows:

(a) the IVS1+63G>A was found in 3/472 (0.64%) chromosomes from ASD patients and in 3/690 (0.43%) chromosomes belonging to 345 Italian controls (patients versus controls, *P *= 0.62)

(b) rs35115415 (c.702G>A) was found in one Italian and one Caucasian-American families (2/472 = 0.42%). This variant was not found in 662 chromosomes belonging to 331 Italian controls. This same variant is reported in dbSNP with an allelic frequency of *A *= 0.02, assessed in 21 Caucasian- and 20 African-Americans from the Coriell Cell Repository collection of apparently healthy individuals

(c) c.872_873delinsAA (S291N) involves the change of contiguous base pairs (TC>AA), present on the same chromosome inherited by the proband from the maternal side in one Caucasian-American family (1/472 = 0.21%). This variant was not found in 650 chromosomes belonging to 325 Italian controls.

**Figure 2:**
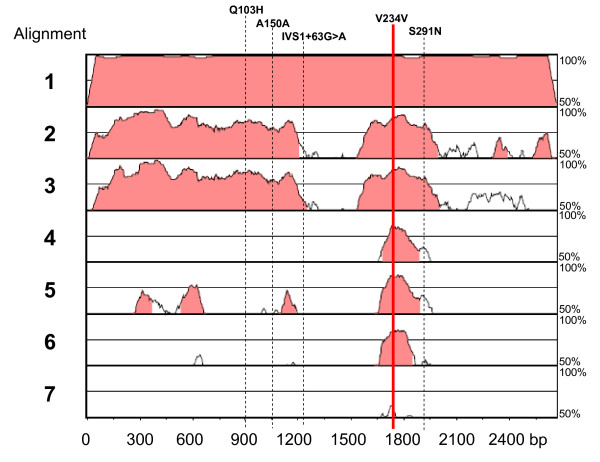
**Phylogenetic conservation analysis by sequence alignment: VISTA plot of the entire *HOXB1 *locus, highlighting the position of SNPs Q103H, A150A, IVS1+63G>A, V234V, and S291N**. Species include Homo sapiens, aligned with (1) *Pan troglodytes*, (2) *Mus musculus*, (3) *Rattus norvegicus*, (4) *Xenopus laevis*, (5) *Gallus gallus*, (6) *Tetraodon nigroviridis *and (7) *Caenorhabditis elegans*, in this order.

**Figure 3:**
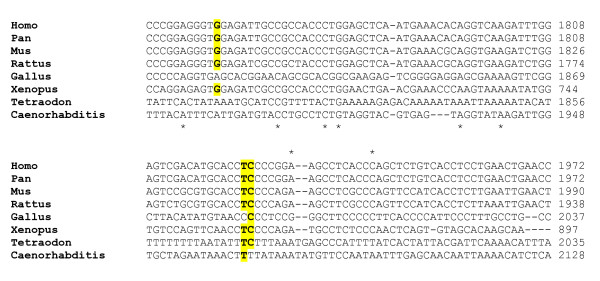
**Phylogenetic conservation analysis by sequence alignment: ClustalW2 output focussed on the V234V (top) and S291N (bottom) SNPs**.

In two families, rare variants IVS1+63G>A and 702G>A (V234V) were transmitted also to an unaffected sibling (Figure 1). Phylogenetic analyses performed using VISTA (Figure 2) and ClustalW2 (Figure 3) demonstrate that especially rs35115415 [c.702G>A (V234V)] and, to some extent, also the S291N rare variants are located in evolutionarily conserved regions.

### Case-control and family based association studies

No significant deviation from HWE was detected by analysing *HOXB1 *common variants in our sample (Table 4). Case-control and family-based association studies were performed using rs72338773 (INS/nINS) and rs12939811 (Q103H); rs7207109 (A150A) was dropped, because it is a synonymous variant in complete linkage disequilibrium with rs12939811 (data not shown). The results of the case-control study are summarized in Table 4. Neither rs72338773 nor rs12939811 show a genotypic or allelic association with autism. Genotypic and allelic distributions in our control sample are very similar to those obtained in 56 CEU individuals (i.e., Utah residents with Northern and Western European ancestry from the CEPH collection) available in public databases [*A/A *= 41 (73.2%), *A/T *= 13 (23.2%), *T/T *= 2 (3.6%); allele *A *= 95 (0.848), allele *T *= 17 (0.152)] and also these do not differ significantly from genotypic and allelic distributions present in our autistic patients [genotype χ^2 ^= 3.65, 2 df, *P *= 0.16; allelic exact *P *= 0.14]. Similarly, a TDT performed on 247 trios, including 172 Italian and 75 Caucasian-American trios, yields a non-significant trend toward the overtransmission of the T309 allele from heterozygous parents to affected offspring (transmitted:non-transmitted = 118:103; χ^2 ^= 1.774, 1 df, *P *= 0.183). In accordance with case-control results, the 9-bp insertion displays an even smaller overtransmission for the INS allele (transmitted: non-transmitted = 127: 118; χ^2 ^= 0.398, 1 df, *P *= 0.528). Collectively, our results do not support an association of large/moderate effect size between autism and HOXB1 gene variants marked by SNPs rs72338773 and rs12939811.

### Phenotypic correlates of HOXB1 gene variants in autism

**Figure 4:**
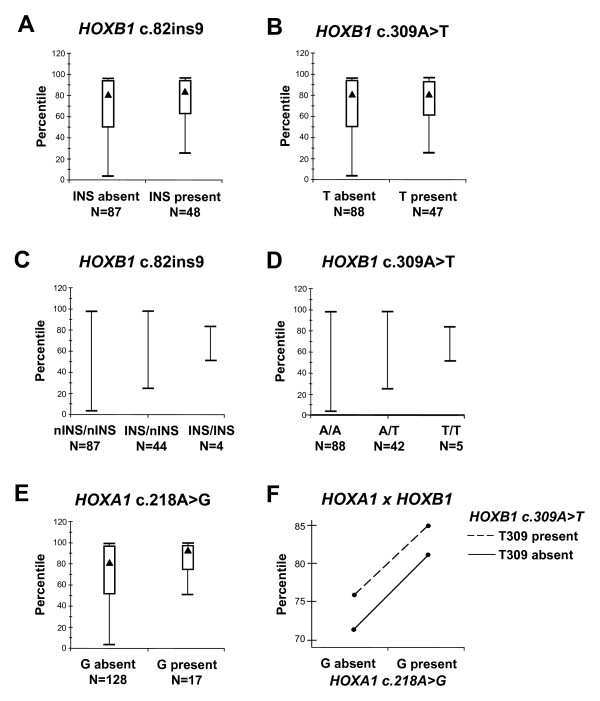
**Cranial circumference and allelic status at SNPs (A, C) *HOXB1 *rs72338773 [c.82insACAGCGCCC (INS/nINS)], (B, D) *HOXB1 *rs12939811 [c.309A>T (Q103H)], (E) *HOXA1 *rs10951154 [c.218A>G (H73R)], and (F) *HOXA1 *× *HOXB1 *interactions at SNPs c.218A>G and c.309A>T, respectively**. Sample sizes are reported for each group below the *X*-axis. Data are expressed as median+interquartilic range (▲ = median; T = I.Q.R.; ▯= non-outliers).

*HOXB1 *alleles exert a modest effect on cranial circumference in autistic patients (Figure 4). Median head sizes are practically superimposable in autistic patients with a presence/absence of INS and/or T309 alleles [INS present versus INS absent = 86.25 ± 17.5 versus 82.5 ± 23.75 percentile, Mann-Whitney (M-W) U = 2609.0, *P *= 0.31; T309 present versus T309 absent = 82.5 ± 17.5 versus 82.5 ± 23.75 percentile, M-W U = 2740.5, *P *= 0.66]. However, autistic patients carrying at least one copy of the INS or T309 alleles display significantly less dispersion in their head circumference distribution compared to patients carrying the nINS/nINS or AA genotypes (Moses test of extreme reactions, *P *< 0.01 for both) (Figure 4, panels A-D). Practically, the lower end of the head circumference range is at the 50th, 25th and 3rd percentile in ASD patients carrying the INS/INS, INS/nINS, and nINS/nINS (or TT, AT and AA) genotype, respectively, and all of the 7/88 (8.0%) ASD patients with head circumferences below the 25th percentile carry the nINS/nINS and AA genotypes (Figure 4, panels C and D). Importantly, these same patients display a much more prominent effect on head size by *HOXA1 *c.218A>G alleles, as previously reported [18,19] (G218 present versus G218 absent = 93.5 ± 16.9 versus 82.5 ± 23.75 percentile, M-W U = 2841.0, *P *= 0.08 and Moses test, *P *< 1 × 10^-7^; (Figure 4, panel E). Finally, we find no evidence of *HOXA1 *× *HOXB1 *interactions, as both appear to independently influence head circumference in autistic patients (Figure 4, panel F).

Analyses exploring possible clinical correlates of *HOXB1 *allelic status in the subset of patients characterized using the ADOS and ADI-R unveiled a possible influence on stereotypic behaviours. Mean total C score at the ADI-R were 7.18 versus 5:30 for 'T309 present' versus 'T309 absent' patients (*N *= 17 and 43, respectively, Student *t *= 2.72, 58 df, *P *< 0.01). This analysis survives Bonferroni correction only when applying the 'relaxed' criterion (see Methods).

## Discussion

The present study describes three rare (IVS1+63G>A, V234V and S291N) and three common (c.82ins9, Q103H and A150A) variants in the *HOXB1 *locus of a total sample of 269 ASD patients. No pathogenetic *de novo *mutation was found as all these gene variants were inherited from one of the parents and at least two are present in other population samples available in public databases. Also, *HOXB1 *common variants do not seem to play major roles in autism pathogenesis, although minor contributions cannot be excluded due to sample size limitations (see below). At the phenotypic level, *HOXB1 *alleles appear to modulate head growth rates to a much lesser extent compared to the *HOXA1 *c.218A>G SNP (Figure 4, compare panels E versus A and B) [18,19]. In reference to cranial circumference, we also find no evidence of gene-gene interaction, since both *HOXA1 *and *HOXB1 *influence head growth independently of each other (Figure 4F). Finally, preliminary analyses involving the subgroup of patients characterized also with the ADI-R suggest that *HOXB1 *alleles may influence stereotypic behaviours. Given the relatively large number of clinical variables assessed for association with *HOXB1 *alleles, this finding survives correction for repeated measures only applying the 'relaxed' criteria, namely those accounting for the non-independence of several variables which are significantly cross-correlated in our sample [38].

In interpreting these results, three limitations of our study design should be considered: (a) we have not specifically assessed for the presence of CNVs encompassing the *HOXB1 *locus in our sample - from a methodological standpoint, DHPLC is not able to distinguish true homozygosity from deletion of one allele; (b) despite employing a significantly larger sample size, compared to previously published reports [17,22-26], our study is still underpowered for common variants in the allelic frequency range of the INS and T309 alleles (see Table 5 at allelic frequencies of 0.2-0.3) and for highly penetrant *de novo *mutations with dominant effects and allelic frequency below 0.02 (Table 6 at an allelic frequency of 0.01); and (c) we cannot evaluate the possible differences in mutation burden at this locus between patients and controls because mutational analysis was performed only among our patient sample.

**Table 5:** Power analysis referring to a family-based design for common variants with low penetrance under an additive model.

Allele frequency	Penetrance			Power
		
	AA	AB	BB	
0.2	0.0	0.2	0.4	0.483

0.3	0.0	0.2	0.4	0.689

0.4	0.0	0.2	0.4	0.800

0.5	0.0	0.2	0.4	0.855

0.6	0.0	0.2	0.4	0.899

0.7	0.0	0.2	0.4	0.902

0.2	0.0	0.15	0.3	0.351

0.3	0.0	0.15	0.3	0.511

0.4	0.0	0.15	0.3	0.637

0.5	0.0	0.15	0.3	0.730

0.6	0.0	0.15	0.3	0.795

0.7	0.0	0.15	0.3	0.735

0.2	0.0	0.1	0.2	0.202

0.3	0.0	0.1	0.2	0.277

0.4	0.0	0.1	0.2	0.396

0.5	0.0	0.1	0.2	0.477

0.6	0.0	0.1	0.2	0.488

0.7	0.0	0.1	0.2	0.505

**Table 6:** Power analysis referring to a family-based design for rare variants with high penetrance and a dominant model.

Allele frequency	Penetrance			Power
		
	AA	AB	BB	
0.10	0.8	0.8	0.0	1.000

0.08	0.8	0.8	0.0	1.000

0.06	0.8	0.8	0.0	1.000

0.04	0.8	0.8	0.0	0.986

0.02	0.8	0.8	0.0	0.642

0.01	0.8	0.8	0.0	0.075

0.10	0.9	0.9	0.0	1.000

0.08	0.9	0.9	0.0	1.000

0.06	0.9	0.9	0.0	1.000

0.04	0.9	0.9	0.0	0.998

0.02	0.9	0.9	0.0	0.767

0.01	0.9	0.9	0.0	0.144

0.10	1.0	1.0	0.0	1.000

0.08	1.0	1.0	0.0	1.000

0.06	1.0	1.0	0.0	1.000

0.04	1.0	1.0	0.0	0.999

0.02	1.0	1.0	0.0	0.751

0.01	1.0	1.0	0.0	0.146

A novel and interesting finding is represented by the location and pattern of inheritance of rare variants. In particular, rs35115415 [c.702G>A (V234V)] and c.872_873delinsAA (S291N) are located in genomic regions which have been evolutionarily conserved from *Tetraodon nigroviridis *and *Caenorhabditis elegans *all the way up to Homo sapiens (Figures 2 and 3). This homeobox domain is probably under high selective pressure and disruptive mutations are probably incompatible with life. Mutations or chromosomal rearrangements involving *HOX *genes and compatible with life are in most cases highly disruptive in animals and in humans, resulting in abnormal limb formation, severe neurological syndromes, leukaemias, or solid tumours [40,41]. In reference to unaffected individuals, publically available databases report one large chorodial neovascularization (CNV; duplication/deletion) involving *HOXB1*, among many other genes, found in two of the 270 individuals of the HapMap collection (see http://projects.tcag.ca/variation/, variation 4040, landmarks chr17:43,957,672... 44,191,836) [42], indicating that the human genome may tolerate some deletions and duplications involving the entire *HOXB1 *locus. However, the *HOXB1 *gene segments hosting rs35115415 [c.702G>A (V234V)] and c.872_873delinsAA (S291N) may possibly accommodate only the less disruptive and non-pathogenic gene variants. Another interesting issue is the parental transmission to the same autistic proband of two distinct rare variants, one transmitted from each parent (Figure 1). Instances where rare variants (single nucleotides and/or CNVs) have no causal effect in heterozygous carriers but are pathogenic in a state of compound heterozygosity are increasingly recognized as a possible cause of autism [43]. However, the rare *HOXB1 *variants described in the present study do not appear pathogenic, as it is biologically implausible that in family T24_IT, for example, the convergence of the intronic variant IVS1+63G>A and the conserved variant 702G>A (V234V) in the same individual may bear dramatic functional consequences. Within the framework of the 'compound heterozygosity' hypothesis, a similar phenomenon should also be occurring in at least one other autism-causing locus. The *a priori *probability of the compound heterozygosity occurs by chance at the *HoxB1 *locus can be estimated at 1.8 × 10^-5 ^and 9.0 × 10^-6 ^for families T24_IT and USA85, respectively. The probability of this same phenomenon occurring by chance at two separate loci in the same family is extraordinarily low, unless boosted by a deficit in genome maintenance mechanisms. Some [44,45], though not all studies [46], support the hypothesis that a small, yet a sizable minority of autism families may display an increased degree of genomic instability. This hypothesis, which is clearly not addressed in the present report, will be the object of further investigation

## Conclusions

In summary, our data indicate that: (a) *HOXB1 *gene variants, either rare or common, do not exert large or moderate effects on affection status in autism spectrum disorders, while minor contributions cannot be excluded due to sample size limitations; (b) modulatory effects on head growth rates are compatible with the developmental roles of this homeobox gene: and (c) *HOXB1 *could influence the clinical autistic phenotype in the area of stereotypic behaviours.

The peculiar coincidence of two distinct rare variants inherited by the autistic probands from both parents in two families raises interest in dysfunctional genome maintenance mechanisms in a subgroup of ASD patients.

## Abbreviations

ADI-R: Autism Diagnostic Interview-Revised; ADOS: Autism Diagnostic Observation Schedule; AGRE: Autism Genetic Resource Exchange; ASD: autism spectrum disorders; CNV: chorodial neovascularization; DHPLC: denaturing high performance liquid chromatography; HWE: Hardy-Weinberg equilibrium; INS: insertion; M-W: Mann-Whitney; PCR: polymerase chain reaction; PDD-NOS: pervasive developmental disorder not otherwise specified; TDT: transmission/disequilibrium test.

## Competing interests

The authors declare that they have no competing interests.

## Authors' contributions

LAM and VG, coordinated by LDA, performed the DHPLCs, genotyping, DNA sequencing and phylogenetic analyses and commented on the manuscript. RS updated the clinical and genetic databases and performed all statistical analyses. Patient recruitment, clinical assessment and sample collections were performed by PC, BM, RA and GG in Rome, RMi and CB in Naples, CL and MS in Milan, CS and RM in Phoenix. AMP contributed to study design, coordinated the recruitment and data collection and drafted the manuscript. All authors read and approved the final manuscript.

## Supplementary Material

Additional file 1 Table S1: Sample sizes involved in the different studies reported here, distinguished by cases/control status and by ethnicity. Click here for file
