# Supplementary material for: Candidate gene study of HOXB1 in autism spectrum disorder
Source: Mol Autism. 2010 May 25;1:9. doi: 10.1186/2040-2392-1-9 (PMC2913946; doi:10.1186/2040-2392-1-9)
Supplement: Additional file 1 — Table S1: Sample sizes involved in the different studies reported here, distinguished by cases/control status and by ethnicity. [file 2040-2392-1-9-S1.DOC]

SUPPLEMENTARY MATERIAL

Supplementary Table S1: Sample sizes involved in the different studies reported here, distinguished by cases/control status and by ethnicity.

|  | Total sample | | Italians | | Caucasian-Americans | |
| --- | --- | --- | --- | --- | --- | --- |
|  | ASD patients | Controls | ASD patients | Controls | ASD patients | Controls |
| N | 269 | 345 | 173 | 345 | 96 | 0 |
| Mutational analysis by DHPLC | 84 | 0 | 84 | 0 | 0 | 0 |
| Case-control association | 169 | 184 | 169 | 184 | 0 | 0 |
| Screening for rare variants | 236 | 325-345 | 173 | 325-345 | 63 | 0 |
| Family-based association | 247 | - | 172 | - | 75 | - |
| Head circumference | 145 | 0 | 145 | 0 | 0 | 0 |
| Stereotypic behaviors | 60 | - | 60 | - | 0 | - |
